# Properties of a subclass of starlike functions involving the quantum derivative operator

**DOI:** 10.1016/j.mex.2025.103740

**Published:** 2025-11-30

**Authors:** D. Breaz, K.R. Karthikeyan, A. Senguttuvan, D. Mohankumar

**Affiliations:** a Department of Mathematics, "1 Decembrie 1918′' University of Alba Iulia, Alba Iulia 510009, Romania; b Department of Applied Mathematics and Science, National University of Science & Technology, Muscat P.O. Box 620, Oman; c Department of Mathematics for Innovation, Saveetha School of Engineering, Saveetha Institute of Medical and Technical Sciences(SIMATS), Thandalam, Chennai 602 105, India

**Keywords:** Analytic function, Univalent function, Schwarz function, Subordination, Coefficient inequalities, Fekete–Szegő inequality, q-hypergeometric function

## Abstract

A new class of functions is defined by expressing analytic characterizations of starlike function involving logarithm. To make this study more versatile, we redefine and study the class involving an operator associated with q-hypergeometric function. Estimates of the initial coefficients and Fekete–Szegő inequality of the functions, which belong to the defined function class, are our main results.

## Highlights

• Classes of analytic functions are defined by using logarithm and a q-differential operator.
• Estimates of $a_{2}$ and $a_{3}$ for functions belonging to the defined function class have been obtained.
• We have obtained the solution to the Fekete–Szegő problem for the defined function classes.

## Graphical abstract

**Figure:**
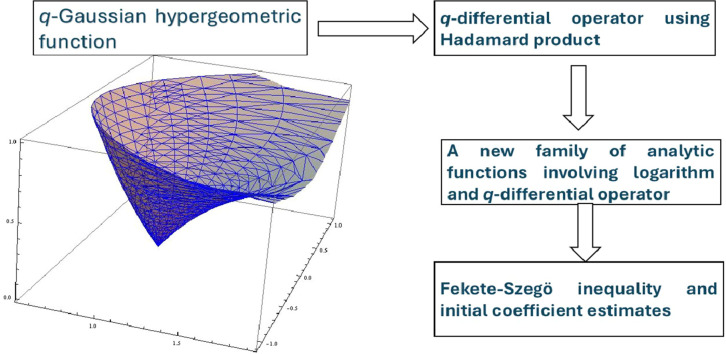

## Specifications table

**Table:** 

**Subject area**	Mathematics and Statistics
**More specific subject area**	Univalent Function Theory In Dual With Special Functions
**Name of your method**	Fekete–Szegő inequality and Logarithmic Coefficients Estimates
**Name and reference of original method**	1. Fekete–Szegő inequality and M. Fekete and G. Szegő, Eine Bemerkung Uber Ungerade Schlichte Funktionen, J. London Math. Soc.**8** (1933), no. 2, 85–89. 2. Logarithmic Coefficients and I. M. Milin, Univalent functions and orthonormal systems, translated from the Russian, Translations of Mathematical Monographs, Vol. 49, Amer. Math. Soc., Providence, RI, 1977.
**Resource availability**	Not applicable.

## Background

Let R,Cand N denote the set of real numbers, set of complex numbers and set of natural numbers respectively. Also let A denote a family of analytic functions defined in Ω={ξ:|ξ|<1} possessing a series of the type

$$\Psi \left(\xi \right)=\xi +\psi_{2}\xi^{2}+\psi_{3}\xi^{3}+\cdots .$$

Here we denote by P, the class of functions of the form $p\left(\xi \right)=1+\sum_{n=1}^{\infty }p_{n}\xi^{n}$ and which satisfies Re(p(ξ)>0,ξ∈Ω. Similary, starlike and convex functions denoted by$\;S^{*}$ and C have the following respective analytic characterizations

$$\frac{\xi \Psi^{\prime }\left(\xi \right)}{\Psi \left(\xi \right)}\in P\;and\;\frac{\left(\xi \Psi^{\prime }\left(\xi \right)\right){}^{\prime }}{\Psi^{{}^{\prime }\left(\xi \right)}}\in P.$$

Ma-Minda [1] studied an analytic function ω∈P which has a series expansion of the form

(1.1) $$\omega \left(\xi \right)=1+M_{1}\xi \;+M_{2}\xi^{2}+M_{3}\xi^{3}+\cdots ,\left(M_{1}>0;\xi \in \Omega \right)$$

studied the following class:

$$S^{*}\left(\omega \right)≔\left\{\Psi \in A:\;\frac{\xi \Psi^{\prime }\left(\xi \right)}{\Psi \left(\xi \right)}≺\omega \left(\xi \right)\right\}$$

and

$$C\left(\omega \right)≔\left\{\Psi \in A:\;\frac{{\left(\xi \Psi^{\prime }\left(\xi \right)\right)}^{\prime }}{\Psi^{{}^{\prime }\left(\xi \right)}}≺\omega \left(\xi \right)\right\},$$

where ≺ denotes the subordination. The classes $S^{*}\left(\omega \right)$and C(ω)was studied by replacing a general function ω(ξ) with a function that maps the unit disc onto a conic regions, for example vertical strip domain [2], shell-like [3,4], three-leaf region [5], Lemniscate of Bernoulli [6,7], Cardioid [8], Crescent [8], Limacon [9] and Nephroid [10].

Lewandowski et al. [11] established the following inclusion

(1.2) $$\;Re\;\left[{\left(\frac{\xi \Psi^{\prime }\left(\xi \right)}{\Psi \left(\xi \right)}\right)}^{\alpha }{\left(\frac{{\left(\xi \Psi^{\prime }\left(\xi \right)\right)}^{{}^{\prime }}}{\Psi^{\prime }\left(\xi \right)}\right)}^{1-\alpha }\right]>0\Rightarrow \Psi \in S^{*},\;\left(\forall \alpha \in R;\Psi \in A\right).$$

The class of functions Ψ∈Asatisfying the differential inequality in (1.2) is known as *alpha starlike function* and here we will denote the class of alpha starlike functions as $L_{\alpha }$.

Motivated by the definition of multiplicative calculus (see [12]), Karthikeyan and Murugusundaramoorthy [13] (see [14]) recently introduced new classes which are defined as follows:

$$R\left(\omega \right)=\left\{\Psi \in A:\;\frac{\xi e^{\frac{\xi^{2}\Psi^{\prime }\left(\xi \right)}{\Psi \left(\xi \right)}}}{\Psi \left(\xi \right)}≺\omega \left(\xi \right),\;\xi \in \Omega \right\}.$$

The classes R(ω) had good geometrical implications. But there is lots of scope for future research, for example establishing the relationship between the various subclasses of univalent functions and the classes introduced involving the multiplicative derivative.

Motivation and Research Objective: Motivated by the classes R(ω) and alpha-starlike function $L_{\alpha }$, we now define a class involving logarithm as follows:

We let $G_{\beta }^{\alpha }\left(\omega \right)$ to denote the class of functions satisfying the conditions

(1.3) $${\left(\frac{\xi \Psi^{\prime }\left(\xi \right)}{\Psi \left(\xi \right)}\right)}^{\alpha }{\left[1+\beta \log\left(\frac{\xi \Psi^{\prime }\left(\xi \right)}{\Psi \left(\xi \right)}\right)\;\right]}^{1-\alpha }≺\omega \left(\xi \right),\;\left(0\leq \alpha \leq 1;\beta \in R\right).$$

where ω∈P has a series expansion of the form (1.2), these powers are considered at the principal branch, and log(.) in (1.3) denotes the single valued branch of the logarithm with log1=0.

Remark 1.1 Needless to point out that for α=0 in$\;G_{\beta }^{\alpha }\left(\omega \right)$, we get the class $G_{\beta }\left(\omega \right)$ which satisfies the condition

$1+\beta \log\left(\frac{\xi \Psi^{\prime }\left(\xi \right)}{\Psi (\xi )}\right)\;≺\omega \left(\xi \right),\;\left(\beta \in R\right).$

**Fig. 1:**
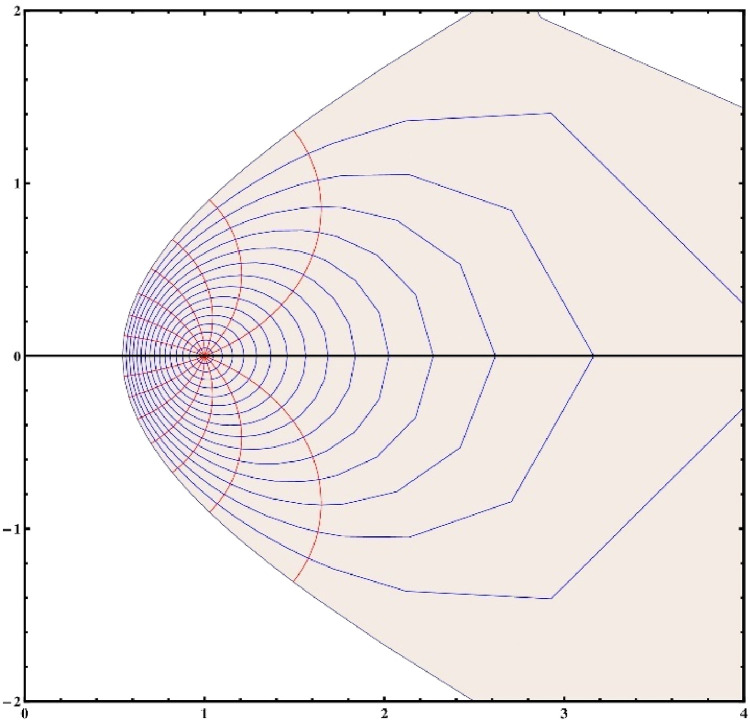
2D image of unit disc under the transformation$\;G\left(\xi \right)={\left(\frac{1}{1-\xi }\right)}^{\frac{1}{4}}{\left[1+\frac{2}{\pi }\log\left(\frac{1}{1-\xi }\right)\;\right]}^{\frac{3}{4}}$.

**Fig. 2:**
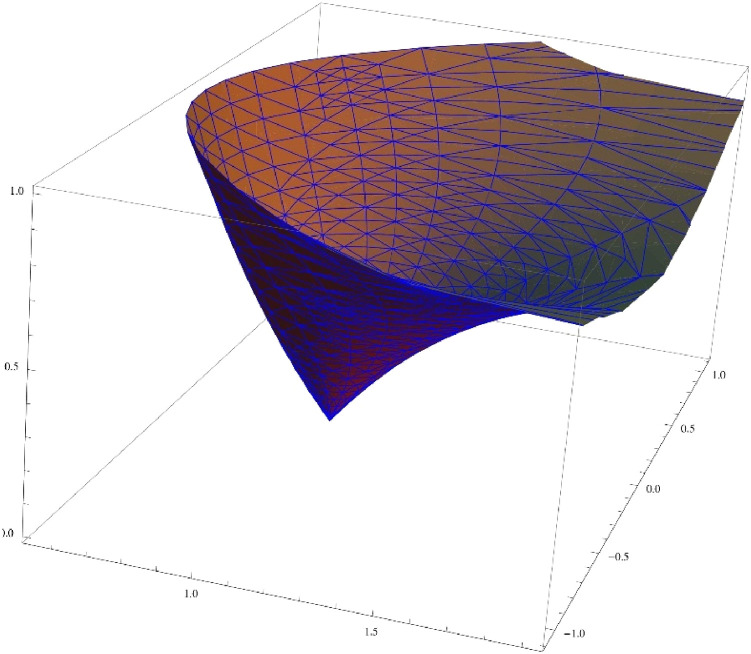
3D image of unit disc under the transformation$\;G\left(\xi \right)={\left(\frac{1}{1-\xi }\right)}^{\frac{1}{4}}{\left[1+\frac{2}{\pi }\log\left(\frac{1}{1-\xi }\right)\;\right]}^{\frac{3}{4}}$.

**Fig. 3:**
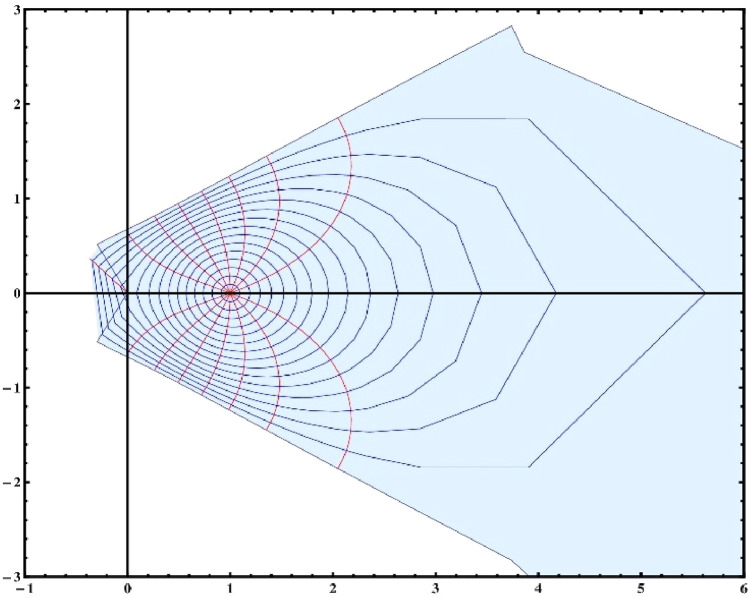
2D image of unit disc under the transformation $H\left(\xi \right)={\left(\frac{1+\xi }{1-\xi }\right)}^{\frac{1}{4}}{\left[1+\frac{2}{\pi }\log\;\left(\frac{1+\xi }{1-\xi }\right)\;\right]}^{\frac{3}{4}}$.

**Fig. 4:**
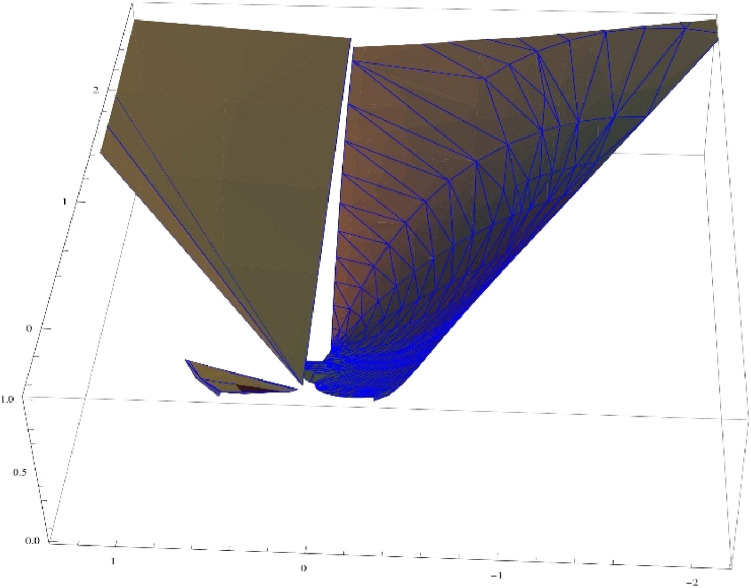
*3*D image of unit disc under the transformation $H\left(\xi \right)={\left(\frac{1+\xi }{1-\xi }\right)}^{\frac{1}{4}}{\left[1+\frac{2}{\pi }\log\;\left(\frac{1+\xi }{1-\xi }\right)\;\right]}^{\frac{3}{4}}$.

Note that for the choice of $\alpha =\frac{1}{4},\;\beta =\frac{2}{\pi }$ and$\;\Psi \left(\xi \right)=\frac{\xi }{1-\xi },$ the Eq. (1.3) reduces to

$$G\left(\xi \right)={\left(\frac{1}{1-\xi }\right)}^{\frac{1}{4}}{\left[1+\frac{2}{\pi }\log\left(\frac{1}{1-\xi }\right)\;\right]}^{\frac{3}{4}}$$

which mapsΩonto a convex domain in the right half plane (see Fig. 1, Fig. 2). Hence the class $G_{\beta }^{\alpha }\left(\omega \right)$ is non-empty. Whereas the Koebe function $\Psi \left(\xi \right)=\frac{\xi }{{\left(1-\xi \right)}^{2}}\;$which is an extremal function of the class $S^{*}$ does not belong to the class $G_{\beta }^{\alpha }\left(\omega \right)$ unless α=0 (see Fig. 3, Fig. 4). Fig. 3, Fig. 4 are the respective 2D and 3D images of unit disc if we let $\alpha =\frac{1}{4},\;\beta =\frac{2}{\pi }$ and$\;\Psi \left(\xi \right)=\frac{\xi }{{\left(1-\xi \right)}^{2}}$ in the left-hand side of (1.3).

We let

$${\left[q\right]}_{n}=\sum_{k=1}^{n}q_{k-1},\;{\left[0\right]}_{q}=0,\;\left(q\in C\right)$$

and the q-shifted factorial by

$${\left(\sigma ;q\right)}_{n}=\left\{ \begin{array}{c} 1,\;n=0\\ \left(1-\sigma \right)\left(1-\sigma q\right)\cdots \left(1-\sigma q^{n-1}\right),\;n=1,\;2,\;\cdots \end{array}\right.$$

For $m\in N_{0}=N\cup \left\{0\right\},\;\delta \geq 0$, $\sigma_{i}\in C\;\left(i-1,2,\cdots ,r\right)$ and $\nu_{i}\in C\setminus Z_{0}^{-};Z_{0}^{-}=\left\{0,\;-1,\cdots \right\}\;\left(j=1,2,\cdots ,s\right),$ Reddy et al. [15] defined the following q-differential operator $Y_{\delta }^{m}\left(\sigma_{1},\;\nu_{1};q,\;\xi \right)\Psi :\Omega \to \Omega$ given by

(1.4) $$Y_{\delta }^{m}\left(\sigma_{1},\;\nu_{1};q,\;\xi \right)\Psi =\xi +\sum_{n=2}^{\infty }{\left[1-\delta +\delta {\left[n\right]}_{q}\right]}^{m}\Gamma_{n}\psi_{n}\xi^{n},$$

where

(1.5) $$\Gamma_{n}=\frac{{\left(\sigma_{1};q\right)}_{n-1\;}{\left(\sigma_{2};q\right)}_{n-1\;}\cdots {\left(\sigma_{r};q\right)}_{n-1\;}}{{\left(q;q\right)}_{n-1\;}{\left(\nu_{1};q\right)}_{n-1\;}\cdots {\left(\nu_{s};q\right)}_{n-1\;}}.$$

Remark 1.2 If we let m=0 in (1.4), then $Y_{\delta }^{m}\left(\sigma_{1},\;\nu_{1};q,\;\xi \right)\Psi$ will reduce to

$$M\left(\sigma_{1},\;\nu_{1};q,\;\xi \right)\Psi =\Psi \left(\xi \right)*G_{r,s}\left(\sigma_{i},\;\nu_{j};q,\;\xi \right)=\xi +\sum_{n=2}^{\infty }\frac{{\left(\sigma_{1};q\right)}_{n-1\;}{\left(\sigma_{2};q\right)}_{n-1\;}\cdots {\left(\sigma_{r};q\right)}_{n-1\;}}{{\left(q;q\right)}_{n-1\;}{\left(\nu_{1};q\right)}_{n-1\;}\cdots {\left(\nu_{s};q\right)}_{n-1\;}}\psi_{n}\xi^{n},$$

where $G_{r,s}\left(\sigma_{i},\;\nu_{j};q,\;\xi \right)=\xi +\sum_{n=2}^{\infty }\frac{{\left(\sigma_{1};q\right)}_{n-1\;}{\left(\sigma_{2};q\right)}_{n-1\;}\cdots {\left(\sigma_{r};q\right)}_{n-1\;}}{{\left(q;q\right)}_{n-1\;}{\left(\nu_{1};q\right)}_{n-1\;}\cdots {\left(\nu_{s};q\right)}_{n-1\;}}\xi^{n}.$ The operator $M(\sigma_{1},\;\nu_{1};q,\;\xi )\Psi$ was introduced by Mohammed and Darus [16] (also see Darus [17]). For discussion on the properties and special cases of the operator${\;Y}_{\delta }^{m}\left(\sigma_{1},\;\nu_{1};q,\;\xi \right)\Psi$, refer to [15,[18], [19], [20]].

## Method details

Now we will redefine the class $G_{\beta }^{\alpha }\left(\omega \right)$ involving the operator $Y_{\delta }^{m}\left(\sigma_{1},\;\nu_{1};q,\;\xi \right)\Psi .$

Definition 2.1 For $\sigma_{i}\in C,\;\left(i=1,2,\cdots ,r\right)$ and $\nu_{1},\nu_{2},\cdots ,\nu_{s},\left(\nu_{j}\in C\setminus Z_{0}^{-};\;Z_{0}^{-}=\left\{0,\;-1,\cdots \right\};\;j=1,2,\cdots ,s;s=r+1\right).\;$Let $C_{\delta }^{m}\left(\sigma_{1},\;\nu_{1};\alpha ,\;\beta ;\omega \right)$ denote the class of functions in A satisfying the inequality

(2.1) $${\left(\frac{\xi {\left[Y_{\delta }^{m}\left(\sigma_{1},\;\nu_{1};q,\;\xi \right)\Psi \left(\xi \right)\right]}^{\prime }}{Y_{\delta }^{m}\left(\sigma_{1},\;\nu_{1};q,\;\xi \right)\Psi \left(\xi \right)}\right)}^{\alpha }\left[1+\beta \log\left(\frac{\xi {\left[Y_{\delta }^{m}\left(\sigma_{1},\;\nu_{1};q,\;\xi \right)\Psi \left(\xi \right)\right]}^{\prime }}{Y_{\delta }^{m}\left(\sigma_{1},\;\nu_{1};q,\;\xi \right)\Psi \left(\xi \right)}\right)\;\right]{\;}^{1-\alpha }≺\omega \left(\xi \right)$$

where 0≤α≤1,β∈R,ω(ξ) be defined as in (1.1), these powers are considered at the principal branch and log(.) in (2.1) denotes the single valued branch of the complex logarithm with log1=0.

Now we will find the solution to the Fekete–Szegő problem for $\Psi \in C_{\delta }^{m}\left(\sigma_{1},\;\nu_{1};\alpha ,\;\beta ;\omega \right)$.

We need the following lemma to establish our main results.

Lemma 2.1 [*21*] *If* $L\left(\xi \right)=1+\sum_{r=1}^{\infty }l_{r}\xi^{r}\in P,\;and\;\varrho \in C,\;then\;$

$$\left|l_{\kappa }-\varrho l_{r}l_{\kappa -r}\right|\leq 2\max\left\{1;\left|2\varrho -1\right|\right\},$$

*for all* 1≤r≤κ−1.

Note that the above results are generalization of the well-known results of Ma-Minda [1] and Livingston [22].

## Method validation

We begin with the following:

Theorem 3.1 *If* $\Psi \left(\xi \right)\in C_{\delta }^{m}\left(\sigma_{1},\;\nu_{1};\alpha ,\;\beta ;\omega \right),\;$*then we have*

(3.1) $$\left|\psi_{2}\right|\leq \frac{M_{1}}{{\left(1+q\delta \right)}^{m}\left[\alpha +\beta -\alpha \beta \right]\left|\Gamma_{2}\right|}$$

(3.2) $$\left|\psi_{3}\right|\leq \frac{M_{1}}{2{\left[1+q\left(q+1\right)\delta \right]}^{m}\left[\alpha +\beta -\alpha \beta \right]\left|\Gamma_{3}\right|}\max\left\{1,\left|\frac{M_{2}}{M_{1}}-\frac{M_{1}\left[\alpha^{2}\left(1-2\beta \right)-3\left(\alpha +\beta \right)+5\alpha \beta \right]}{2{\left[\alpha +\beta -\alpha \beta \right]}^{2}}\right|\;\right\}$$

*and for all* ρ∈C*,*

(3.3) $$\left|\psi_{3}-\rho \psi_{2}^{2}\right|\leq \frac{M_{1}}{2{\left[1+q\left(q+1\right)\delta \right]}^{m}\left[\alpha +\beta -\alpha \beta \right]\left|\Gamma_{3}\right|}\max\left\{1,\left|\frac{M_{2}}{M_{1}}-\frac{M_{1}\left[\alpha^{2}\left(1-2\beta \right)-3\left(\alpha +\beta \right)+5\alpha \beta \right]}{2{\left[\alpha +\beta -\alpha \beta \right]}^{2}}-\frac{2\rho M_{1}{\left[1+q\left(q+1\right)\delta \right]}^{m}\left[\alpha +\beta -\alpha \beta \right]\Gamma_{3}}{{\left(1+q\delta \right)}^{2m}{\left[\alpha +\beta -\alpha \beta \right]}^{2}\Gamma_{2}^{2}}\;\right|\right\},$$

*where* $\Gamma_{2}$ *and* $\Gamma_{3}$ *are defined as in* (1.5).

***Proof.*** As $\Psi \left(\xi \right)\in C_{\delta }^{m}\left(\sigma_{1},\;\nu_{1};\alpha ,\;\beta ;\omega \right)$, by (2.1), we have

(3.4) $${\left(\frac{\xi {\left[Y_{\delta }^{m}\left(\sigma_{1},\;\nu_{1};q,\;\xi \right)\Psi \left(\xi \right)\right]}^{\prime }}{Y_{\delta }^{m}\left(\sigma_{1},\;\nu_{1};q,\;\xi \right)\Psi \left(\xi \right)}\right)}^{\alpha }\left[1+\beta \log\left(\frac{\xi {\left[Y_{\delta }^{m}\left(\sigma_{1},\;\nu_{1};q,\;\xi \right)\Psi \left(\xi \right)\right]}^{\prime }}{Y_{\delta }^{m}\left(\sigma_{1},\;\nu_{1};q,\;\xi \right)\Psi \left(\xi \right)}\right)\;\right]{\;}^{1-\alpha }=\omega \left[w\left(\psi \right)\right].$$

Clearly, ϑ of the form $\vartheta \left(\xi \right)=1+\sum_{r=1}^{\infty }\vartheta_{n}\xi^{n}\in P,\;and\;is\;defined\;by\;$

$\vartheta \left(\xi \right)=\frac{1+w(\psi )}{1-w(\xi )},\;\xi \in \Omega .$

The right side of (3.4) will be of the form

(3.5) $$\omega \left[w\left(\psi \right)\right]=1+\frac{\vartheta_{1}M_{1}}{2}\xi +\frac{M_{1}}{2}\left[\vartheta_{2}-\frac{\vartheta_{1}^{2}}{2}\left(1-\frac{M_{2}}{M_{1}}\right)\right]\xi^{2}+\cdots .$$

The left-hand side of (3.4) will be of the form

(3.6) $${\left(\frac{\xi {\left[Y_{\delta }^{m}\left(\sigma_{1},\;\nu_{1};q,\;\xi \right)\Psi \left(\xi \right)\right]}^{\prime }}{Y_{\delta }^{m}\left(\sigma_{1},\;\nu_{1};q,\;\xi \right)\Psi \left(\xi \right)}\right)}^{\alpha }\left[1+\beta \log\left(\frac{\xi {\left[Y_{\delta }^{m}\left(\sigma_{1},\;\nu_{1};q,\;\xi \right)\Psi \left(\xi \right)\right]}^{\prime }}{Y_{\delta }^{m}\left(\sigma_{1},\;\nu_{1};q,\;\xi \right)\Psi \left(\xi \right)}\right)\;\right]{\;}^{1-\alpha }$$

$=1+\left[\alpha +\beta -\alpha \beta \right]\psi_{2}\xi +\frac{1}{2}\{4\psi_{3}\left(\alpha +\beta -\alpha \beta \right)+\psi_{2}^{2}\left[\alpha^{2}\left(1-2\beta \right)-3\left(\alpha +\beta \right)-5-\alpha \beta \right]\xi^{2}+\cdots .$

From (3.6) and (3.5), we obtain

(3.7) $$\psi_{2}=\frac{\vartheta_{1}M_{1}}{2\left(1+q\delta \right)\left[\alpha +\beta -\alpha \beta \right]\Gamma_{2}}$$

and

(3.8) $$\psi_{3}=\frac{M_{1}}{4\left[1+q\left(q+1\right)\delta \right]\left[\alpha +\beta -\alpha \beta \right]\Gamma_{3}}\left[\vartheta_{2}-\frac{\vartheta_{1}^{2}}{2}\left(1-\frac{M_{2}}{M_{1}}+\frac{M_{1}\left[\alpha^{2}\left(1-2\beta \right)-3\left(\alpha +\beta \right)+5\alpha \beta \right]}{2{\left[\alpha +\beta -\alpha \beta \right]}^{2}}\;\right)\right].$$

Equations (3.1) can be obtained by applying $|\vartheta_{1}|\leq 2$ ([23]) in (3.7). Using Lemma 2.1 in (3.8), we get (3.2).

Now to prove (3.3), we consider

$$\left|\psi_{3}-\rho \psi_{2}^{2}\right|=\left|\frac{M_{1}}{4\left[1+q\left(q+1\right)\delta \right]\left[\alpha +\beta -\alpha \beta \right]\Gamma_{3}}\left[\vartheta_{2}-\frac{\vartheta_{1}^{2}}{2}\left(1-\frac{M_{2}}{M_{1}}+\frac{M_{1}\left[\alpha^{2}\left(1-2\beta \right)-3\left(\alpha +\beta \right)+5\alpha \beta \right]}{2{\left[\alpha +\beta -\alpha \beta \right]}^{2}}\;\right)\right]-\frac{\rho \vartheta_{1}^{2}M_{1}^{2}}{4{\left(1+q\delta \right)}^{2}{\left[\alpha +\beta -\alpha \beta \right]}^{2}\Gamma_{2}^{2}}\right|.$$

$$\;=\left|\frac{M_{1}}{4\left[1+q\left(q+1\right)\delta \right]\left[\alpha +\beta -\alpha \beta \right]\Gamma_{3}}\left[\vartheta_{2}-\frac{\vartheta_{1}^{2}}{2}\left(1-\frac{M_{2}}{M_{1}}+\frac{M_{1}\left[\alpha^{2}\left(1-2\beta \right)-3\left(\alpha +\beta \right)+5\alpha \beta \right]}{2{\left[\alpha +\beta -\alpha \beta \right]}^{2}}+\frac{2\rho M_{1}\left[1+q\left(q+1\right)\delta \right]\left[\alpha +\beta -\alpha \beta \right]\Gamma_{3}}{{\left(1+q\delta \right)}^{2}{\left[\alpha +\beta -\alpha \beta \right]}^{2}\Gamma_{2}^{2}}\;\right)\right]\right|.$$

Using Lemma 2.1 in the above equation, we get the result (3.3).

Letting $r=2,\;s=1,\sigma_{1}=\nu_{1},\;\sigma_{2}=q,\;m=0\;\text{and}\;q\to 1-$ in Theorem 3.1, we get

Corollary 3.2 *If* $\Psi \left(\xi \right)\in G_{\beta }^{\alpha }\left(\omega \right),\;then\;we\;have$

$$\left|\psi_{2}\right|\leq \frac{M_{1}}{\left[\alpha +\beta -\alpha \beta \right]}$$

$$\left|\psi_{3}\right|\leq \frac{M_{1}}{2\left[\alpha +\beta -\alpha \beta \right]}\max\left\{1,\left|\frac{M_{2}}{M_{1}}-\frac{M_{1}\left[\alpha^{2}\left(1-2\beta \right)-3\left(\alpha +\beta \right)+5\alpha \beta \right]}{2{\left[\alpha +\beta -\alpha \beta \right]}^{2}}\right|\;\right\}$$

*and for all* ρ∈C

$$\left|\psi_{3}-\rho \psi_{2}^{2}\right|\leq \frac{M_{1}}{2\left[\alpha +\beta -\alpha \beta \right]}\max\left\{1,\left|\frac{M_{2}}{M_{1}}-\frac{M_{1}\left[\alpha^{2}\left(1-2\beta \right)-3\left(\alpha +\beta \right)+5\alpha \beta \right]}{2{\left[\alpha +\beta -\alpha \beta \right]}^{2}}-\frac{2\rho M_{1}\left[\alpha +\beta -\alpha \beta \right]}{{\left[\alpha +\beta -\alpha \beta \right]}^{2}}\;\right|\right\}.$$

Letting α=1andq→1− in Corollary 3.2, we get

Corollary 3.3 *If* $\Psi \left(\xi \right)\in S^{*}\left(\omega \right),\;then\;we\;have$

$$\left|\psi_{2}\right|\leq M_{1},\;\left|\psi_{3}\right|\leq M_{1}\max\left\{1,\left|\frac{M_{2}}{M_{1}}+M_{1}\right|\;\right\}$$

*and for a complex number* ρ,

$$\left|\psi_{3}-\rho \psi_{2}^{2}\right|\leq \frac{M_{1}}{2}\max\left\{1,\left|\frac{M_{2}}{M_{1}}+M_{1}\left(1-2\rho \right)\right|\right\}.$$

Setting $r=2,\;s=1,\sigma_{1}=\nu_{2},\;\sigma_{2}=q,\;m=0,\;\alpha =1\;\text{and}\;q\to 1-$ in Theorem 3.1, we get

Corollary 3.4 *If* Ψ(ξ)∈C(ω),then

$$\left|\psi_{2}\right|\leq \frac{M_{1}}{2},\;\left|\psi_{3}\right|\leq \frac{M_{1}}{6}\max\left\{1,\left|\frac{M_{2}}{M_{1}}+M_{1}\right|\;\right\}$$

*and for a complex number* ρ,

$$\left|\psi_{3}-\rho \psi_{2}^{2}\right|\leq \frac{M_{1}}{6}\max\left\{1,\left|\frac{M_{2}}{M_{1}}+M_{1}\left(1-\frac{3\rho }{2}\right)\right|\right\}.$$

Letting $\omega \left(\xi \right)=\frac{1+\xi }{1-\xi }$ in Corollaries 3.3 and 3.4, we get

Corollary 3.5 *([**24**], [**25**]) If* $\Psi \in S^{*},\;$*then,*

$$\left|\psi_{2}\right|\leq 2,\;\left|\psi_{3}\right|\leq 3$$

*and for a complex number* ρ,

$$\left|\psi_{3}-\rho \psi_{2}^{2}\right|\leq \max\left\{1,\left|3-4\rho \right|\right\}.$$

The Fekete–Szegő inequality of $S^{*}$ also appears in the proof of such an inequality for the class of close-to-convex function [25].

Corollary 3.6 *([**24**], [**25**]) If* Ψ∈C,then*,*

$$\left|\psi_{2}\right|\leq 1,\;\left|\psi_{3}\right|\leq 1$$

*and for a complex number* ρ,

$$\left|\psi_{3}-\rho \psi_{2}^{2}\right|\leq \frac{1}{3}\max\left\{1,\;3\left|1-\rho \right|\right\}.$$

Setting $r=2,\;s=1,\sigma_{1}=\nu_{1},\;\sigma_{2}=q,\;m=1,\;\alpha =1\;\text{and}\;q\to 1-$ in Theorem 3.1, we get

Corollary 3.7 *If* Ψ∈MC(ω),then*,*

$$\left|\psi_{2}\right|\leq \frac{M_{1}}{\left(1+\delta \right)},\;\left|\psi_{3}\right|\leq \frac{M_{1}}{2\left(1+2\delta \right)}\max\left\{1,\left|\frac{M_{2}}{M_{1}}+M_{1}\right|\;\right\}$$

*and for a complex number* ρ,

$$\left|\psi_{3}-\rho \psi_{2}^{2}\right|\leq \frac{M_{1}}{2\left(1+2\delta \right)}\max\left\{1,\left|\frac{M_{2}}{M_{1}}+M_{1}\left(1-\frac{2\rho \left(1+2\delta \right)}{{\left(1+\delta \right)}^{2}}\right)\right|\right\}.$$

Corollary 3.8 *([*[26], [27], [28]*]) If* $\Psi \in MC(\frac{1+\xi }{1-\xi }),\;then$

$$\left|\psi_{2}\right|\leq \frac{2}{\left(1+\delta \right)},\;\left|\psi_{3}\right|\leq \frac{3}{\left(1+2\delta \right)},\;\left|\psi_{3}-\rho \psi_{2}^{2}\right|\leq \frac{1}{\left(1+2\delta \right)}\max\left\{1,\left|3-\frac{4\rho \left(1+2\delta \right)}{{\left(1+\delta \right)}^{2}}\right|\right\}.$$

*where* ρ *is a complex number.*

Remark 3.1 Setting δ=1andδ=1 in Corollary 3.7, we can get corollaries 3.3 and 3.4 respectively.

Now we will proceed to find the coefficient estimates of $\Psi^{-1}\left(\xi \right)$. The inverse $\Psi^{-1},$ defined by $\Psi^{-1}\left(\Psi (\xi )\right)=\xi ,\;\xi \in \Omega$ and $\Psi^{-1}\left(\Psi (t)\right)=t,\;\left(|t|<r;r\geq \frac{1}{4}\right)$ where

(4.1) $$g\left(t\right)=\Psi^{-1}\left(t\right)=t-\psi_{2}t^{2}+\left(2\psi_{2}^{2}-\psi_{3}\right)t^{3}-\left(5\psi_{2}^{2}-5\psi_{2}\psi_{3}+\psi_{4}\right)t^{4}+\cdots .$$

The coefficient inequalities of the inverse functions $C_{\delta }^{m}\left(\sigma_{1},\;\nu_{1};\alpha ,\;\beta ;\omega \right)$ are valid only for the functions which are univalent.

Theorem 4.1 *Let* $\Psi \in C_{\delta }^{m}\left(\sigma_{1},\;\nu_{1};\alpha ,\;\beta ;\omega \right)$*and let* $\Psi^{-1}$*be the inverse of* Ψ*defined by*

$$\Psi^{-1}\left(t\right)=w+\sum_{k=2}^{\infty }\chi_{k}t^{k},\;\left(\left|t\right|<r;r\geq \frac{1}{4}\right),\;$$

*then for* β≠1, *we have*

$$\;\left|\chi_{2}\right|\leq \frac{M_{1}}{{\left(1+q\delta \right)}^{m}\left[\alpha +\beta -\alpha \beta \right]\left|\Gamma_{2}\right|}$$

*and*

$|\chi_{3}|\leq \frac{M_{1}}{2{\left[1+q(q+1)\delta \right]}^{m}\left[\alpha +\beta -\alpha \beta \right]\left|\Gamma_{3}\right|}\max\left\{1,|\frac{M_{2}}{M_{1}}-\frac{M_{1}\left[\alpha^{2}\left(1-2\beta \right)-3\left(\alpha +\beta \right)+5\alpha \beta \right]}{2{\left[\alpha +\beta -\alpha \beta \right]}^{2}}-\frac{4M_{1}{\left[1+q(q+1)\delta \right]}^{m}\left[\alpha +\beta -\alpha \beta \right]\Gamma_{3}}{{\left(1+q\delta \right)}^{2m}{\left[\alpha +\beta -\alpha \beta \right]}^{2}\Gamma_{2}^{2}}|\;\right\}.$

Also for all τ∈C

$$\left|\chi_{3}-\tau \chi_{2}^{2}\right|\leq \frac{M_{1}}{2{\left[1+q\left(q+1\right)\delta \right]}^{m}\left[\alpha +\beta -\alpha \beta \right]\left|\Gamma_{3}\right|}\max\left\{1,\left|\frac{M_{2}}{M_{1}}-\frac{M_{1}\left[\alpha^{2}\left(1-2\beta \right)-3\left(\alpha +\beta \right)+5\alpha \beta \right]}{2{\left[\alpha +\beta -\alpha \beta \right]}^{2}}-\frac{2\left(\tau -2\right)M_{1}{\left[1+q\left(q+1\right)\delta \right]}^{m}\left[\alpha +\beta -\alpha \beta \right]\Gamma_{3}}{{\left(1+q\delta \right)}^{2m}{\left[\alpha +\beta -\alpha \beta \right]}^{2}\Gamma_{2}^{2}}\;\right|\right\},$$

*where* $\Gamma_{2}$ *and* $\Gamma_{3}$ *are defined as in (*1.5*).*

***Proof.*** From $\Psi \left(\xi \right)=\xi +\sum_{n=2}^{\infty }\psi_{n}\xi^{n}$ and (4.1), we have

$\chi_{2}=-\psi_{2}\;and\;\chi_{3}=2\psi_{2}^{2}-\psi_{3}.$

The estimate for $|\chi_{2}\left|=\right|\psi_{2}|$ can be got by taking modulus of (3.7). Letting ρ=2in (3.3), we get $|\chi_{3}|.\;$To find the Fekete–Szegő inequality for the inverse function, consider

$|\chi_{3}-\tau \chi_{2}^{2}\left|=\right|2\psi_{2}^{2}-\psi_{3}-\tau \psi_{2}^{2}\left|=\right|\psi_{3}-\left(\tau -2\right)\psi_{2}^{2}|.$

Changing ρ=(τ−2) in the (3.3), we get the desired result.

We will now proceed to find logarithmic coefficients for functions belonging to $C_{\delta }^{m}\left(\sigma_{1},\;\nu_{1};\alpha ,\;\beta ;\omega \right)$. Logarithmic coefficients took the spotlight when Milin in [29] studied its properties which would imply the bounds of the Taylor coefficients of univalent functions. Refer to [[30], [31], [32]] for the detailed study on properties and significance of the logarithmic coefficients.

If Ψ is analytic in Ω, with $\frac{\Psi (\xi )}{\xi }\neq 0$ for all ξ∈Ω, then the well-known logarithmic coefficients $\phi_{n}=\phi_{n}\left(\psi \right),\;n\in N$, of Ψ are given by

(5.1) $$\log\frac{\Psi \left(\xi \right)}{\xi }=2\sum_{n=1}^{\infty }\phi_{n}\xi^{n},\;\xi \in \Omega ,\;\log1=0.$$

Now we will add additional criterion to the class $C_{\delta }^{m}\left(\sigma_{1},\;\nu_{1};\alpha ,\;\beta ;\omega \right),\;$so that logarithmic coefficients of $C_{\delta }^{m}\left(\sigma_{1},\;\nu_{1};\alpha ,\;\beta ;\omega \right)$ is well-defined. That is, we let $BC_{\delta }^{m}\left(\sigma_{1},\;\nu_{1};\alpha ,\;\beta ;\omega \right)=C_{\delta }^{m}\left(\sigma_{1},\;\nu_{1};\alpha ,\;\beta ;\omega \right)\cap \left\{\Psi \;is\;analytic\;in\;\Omega :\frac{\Psi (\xi )}{\xi }\neq 0,\;\xi \in \Omega \right\}$. Note that for all functions $BC_{\delta }^{m}\left(\sigma_{1},\;\nu_{1};\alpha ,\;\beta ;\omega \right)$, the relation (5.1) is well-defined.

Theorem 5.1 *If* $\Psi \left(\xi \right)\in BC_{\delta }^{m}\left(\sigma_{1},\;\nu_{1};\alpha ,\;\beta ;\omega \right)$ *with the logarithmic coefficients given by (*5.1*), then for* β≠1, *we have*

$$\left|\phi_{1}\right|\leq \frac{M_{1}}{2{\left(1+q\delta \right)}^{m}\left[\alpha +\beta -\alpha \beta \right]\left|\Gamma_{2}\right|}\;,$$

$$\left|\phi_{2}\right|\leq \frac{M_{1}}{4{\left[1+q\left(q+1\right)\delta \right]}^{m}\left[\alpha +\beta -\alpha \beta \right]\left|\Gamma_{3}\right|}\max\left\{1,\left|\frac{M_{2}}{M_{1}}-\frac{M_{1}\left[\alpha^{2}\left(1-2\beta \right)-3\left(\alpha +\beta \right)+5\alpha \beta \right]}{2{\left[\alpha +\beta -\alpha \beta \right]}^{2}}-\frac{M_{1}{\left[1+q\left(q+1\right)\delta \right]}^{m}\left[\alpha +\beta -\alpha \beta \right]\Gamma_{3}}{{\left(1+q\delta \right)}^{2m}{\left[\alpha +\beta -\alpha \beta \right]}^{2}\Gamma_{2}^{2}}\right|\;\right\},$$

$$\left|\phi_{2}-\mu \phi_{1}^{2}\right|\leq \frac{M_{1}}{4{\left[1+q\left(q+1\right)\delta \right]}^{m}\left[\alpha +\beta -\alpha \beta \right]\left|\Gamma_{3}\right|}\max\left\{1,\left|\frac{M_{2}}{M_{1}}-\frac{M_{1}\left[\alpha^{2}\left(1-2\beta \right)-3\left(\alpha +\beta \right)+5\alpha \beta \right]}{2{\left[\alpha +\beta -\alpha \beta \right]}^{2}}-\frac{\left(1+\mu \right)M_{1}{\left[1+q\left(q+1\right)\delta \right]}^{m}\left[\alpha +\beta -\alpha \beta \right]\Gamma_{3}}{{\left(1+q\delta \right)}^{2m}{\left[\alpha +\beta -\alpha \beta \right]}^{2}\Gamma_{2}^{2}}\;\right|\right\},$$

*where* $\Gamma_{2}$ *and* $\Gamma_{3}$ *are defined as in (*1.5*).*

***Proof.*** *From* $\Psi \left(\xi \right)=\xi +\sum_{n=2}^{\infty }\psi_{n}\xi^{n}$and equating the first two coefficients or relation (5.1), we get

$\phi_{1}=\frac{\psi_{2}}{2},\;\phi_{2}=\frac{1}{2}\left(\psi_{3}-\frac{\psi_{2}^{2}}{2}\right).$

Using (3.7) and (3.8) in the above equation and applying Lemma 2.1, we obtain the bounds of $|\phi_{1}|$ and $|\phi_{2}|$. To obtain the bound of $|\phi_{2}-\mu \phi_{1}^{2}|$, consider

$|\phi_{2}-\mu \phi_{1}^{2}|=\frac{1}{2}\left|\psi_{3}-\frac{\left(1+\mu \right)}{2}\psi_{1}^{2}\right|.$

Changing $\rho =\frac{1+\mu }{2}$ in (3.3), we get the desired result.

To unify and discretize the results obtained so far, we will now define a subclass of analytic functions with respect to symmetric points. Quantum derivative is a concept motivated by Newton’s divided difference rule used to find the approximate value of the derivative at a fixed point, is just a ratio which avoids limits concept which is an important ingredient in the traditional calculus. Since quantum calculus is very well-known, we will avoid discussing its formal definition, motivation and its applications here. The textbook by Annaby and Mansour [33] would serve as a single source of reference pertaining to quantum calculus require to study this section.

For Ψ∈A and 0<q<1, the q-difference operator for a function f∈A is defined by

(6.1) $$D_{q}\Psi \left(\xi \right)=\left\{ \begin{array}{c} \Psi^{\prime }\left(0\right),\;if\;\xi =0\;\\ \frac{\Psi \left(\xi \right)-\Psi \left(q\xi \right)}{\left(1-q\right)\xi },\;if\;\xi \neq 0 \end{array}\right.$$

In this section, we will define a new family Sakaguchi type analytic functions using quantum derivative. Sakaguchi [34] considered a family $S_{s}^{*}$ of starlike functions with respect to symmetrical points, by defining the family

$S_{s}^{*}=\left\{\Psi \in A:\;Re\frac{2\xi \Psi^{\prime }\left(\xi \right)}{\Psi (\xi )-\Psi (-\xi )}>0,\;\xi \in \Omega \right\}.$

Motivated by the Sakaguchi class $S_{s}^{*},$ we will define the following:

Definition 6.1 For $\sigma_{i}\in C,\;\left(\;i=1,2,\cdots r\right)\;and\;\nu_{1},\;\nu_{2},\cdots ,\;\nu_{s},\;\left(\nu_{j}\in C\setminus Z_{0}^{-};\;Z_{0}^{-}=\left\{0,\;-1,\;-2,\cdots \right\};j=1,2,\cdots ,s;r=s+1\right).\;$Let $SC_{\delta }^{m}\left(\sigma_{1},\;\nu_{1};\alpha ,\;\beta ;\omega \right)$ denote the class of functions in A satisfying the inequality

(6.2) $${\left(\frac{2\xi D_{q}\left[Y_{\delta }^{m}\left(\sigma_{1},\;\nu_{1};q,\;\xi \right)\Psi \left(\xi \right)\right]}{H\left(\xi \right)}\right)}^{\alpha }\left[1+\beta \log\left(\frac{2\xi D_{q}\left[Y_{\delta }^{m}\left(\sigma_{1},\;\nu_{1};q,\;\xi \right)\Psi \left(\xi \right)\right]}{H\left(\xi \right)}\right)\;\right]{\;}^{1-\alpha }≺\omega \left(\xi \right)$$

Where $H\left(\xi \right)=Y_{\delta }^{m}\left(\sigma_{1},\;\nu_{1};q,\;\xi \right)\Psi \left(\xi \right)-Y_{\delta }^{m}\left(\sigma_{1},\;\nu_{1};q,\;\xi \right)\Psi \left(-\xi \right),\;0\leq \alpha \leq 1,\;\beta \in R,\;\omega \left(\xi \right)$ be defined as in (1.1) and these powers are considered at the main branch, that is log1=0.

In this section, we will present only the results whose proof is analogous to those obtained in the preceding sections.

Theorem 6.1 *If* $\Psi \left(\xi \right)\in SC_{\delta }^{m}\left(\sigma_{1},\;\nu_{1};\alpha ,\;\beta ;\omega \right),\;$*then we have*

$$\left|\psi_{2}\right|\leq \frac{M_{1}}{\left|{\left[2\right]}_{q}\left[\alpha +\beta -\alpha \beta \right]R_{2}\right|}$$

$$\left|\psi_{3}\right|\leq \frac{M_{1}}{\left|\left({\left[3\right]}_{q}-1\right)\left[\alpha +\beta -\alpha \beta \right]R_{3}\right|}\max\left\{1,\left|\frac{M_{2}}{M_{1}}+\frac{M_{1}\left[\alpha^{2}\left(\beta -1\right)+\alpha +\beta \left(1-3\alpha \right)\right]}{2{\left[\alpha +\beta -\alpha \beta \right]}^{2}}\right|\right\}$$

*and for all* ρ∈C

$$\left|\psi_{3}-\rho \psi_{2}^{2}\right|\leq \frac{M_{1}}{\left|\left({\left[3\right]}_{q}-1\right)\left[\alpha +\beta -\alpha \beta \right]R_{3}\right|}\max\left\{1,\left|\frac{M_{2}}{M_{1}}+\frac{M_{1}\left[\alpha^{2}\left(\beta -1\right)+\alpha +\beta \left(1-3\alpha \right)\right]}{2{\left[\alpha +\beta -\alpha \beta \right]}^{2}}-\frac{\rho M_{1}\left({\left[3\right]}_{q}-1\right)R_{3}}{{\left[2\right]}_{q}^{2}{\left[\alpha +\beta -\alpha \beta \right]}^{2}R_{2}^{2}}\right|\right\},$$

where$\;R_{n}=\left[1-\delta +\delta {\left[n\right]}_{q}^{m}\right]\frac{{\left(\sigma_{1};q\right)}_{n-1\;}{\left(\sigma_{2};q\right)}_{n-1\;}\cdots {\left(\sigma_{r};q\right)}_{n-1\;}}{{\left(q;q\right)}_{n-1\;}{\left(\nu_{1};q\right)}_{n-1\;}\cdots {\left(\nu_{s};q\right)}_{n-1\;}}.$

Theorem 6.2 *Let* $\Psi \in SC_{\delta }^{m}\left(\sigma_{1},\;\nu_{1};\alpha ,\;\beta ;\omega \right)$*and let* $\Psi^{-1}$*be the inverse of* Ψ*defined by*

$$\Psi^{-1}\left(t\right)=w+\sum_{k=2}^{\infty }\chi_{k}t^{k},\;\left(\left|t\right|<r;r\geq \frac{1}{4}\right),\;$$

*then for* β≠1, *we have*

$$\left|\chi_{2}\right|\leq \frac{M_{1}}{\left|{\left[2\right]}_{q}\left[\alpha +\beta -\alpha \beta \right]R_{2}\right|}$$

*and*

$|\chi_{3}|\leq \frac{M_{1}}{|\left({\left[3\right]}_{q}-1\right)\left[\alpha +\beta -\alpha \beta \right]R_{3}|}\max\left\{1,|\frac{M_{2}}{M_{1}}+\frac{M_{1}\left[\alpha^{2}\left(\beta -1\right)+\alpha +\beta \left(1-3\alpha \right)\right]}{2{\left[\alpha +\beta -\alpha \beta \right]}^{2}}-\frac{2M_{1}\left({\left[3\right]}_{q}-1\right)R_{3}}{{\left[2\right]}_{q}^{2}{\left[\alpha +\beta -\alpha \beta \right]}^{2}R_{2}^{2}}|\;\right\}.$

Also for all τ∈C

$$\left|\chi_{3}-\tau \chi_{2}^{2}\right|\leq \frac{M_{1}}{\left|\left({\left[3\right]}_{q}-1\right)\left[\alpha +\beta -\alpha \beta \right]R_{3}\right|}\max\left\{1,\left|\frac{M_{2}}{M_{1}}+\frac{M_{1}\left[\alpha^{2}\left(\beta -1\right)+\alpha +\beta \left(1-3\alpha \right)\right]}{2{\left[\alpha +\beta -\alpha \beta \right]}^{2}}-\frac{\left(\tau -2\right)M_{1}\left({\left[3\right]}_{q}-1\right)R_{3}}{{\left[2\right]}_{q}^{2}{\left[\alpha +\beta -\alpha \beta \right]}^{2}R_{2}^{2}}\;\right|\right\}.$$

***Concluding remarks and observations:*** As a concluding remark, here we summarize our primary contribution and further scope of research. Motivated by the characterization of gamma starlike function, here we have introduced and studied a new family which was expressed as combination of differential characterizations belonging to starlike and logarithm of starlike functions. We obtained bounds of the initial coefficients of functions belonging to the defined function classes. Since the defined function class is subordinate to a general function and involves lots of parameters, our main results have lots of applications. Further scope on findings of the methods studied here would be explore the behavior after replacing the classical derivative with a non-Newtonian derivative [35].

## Related research article

Zdzisław Lewandowski, Sanford S. Miller, and Eligiusz Złotkiewicz Gamma-starlike functions. Ann. Univ. Mariae Curie-Skłodowska Sect. A, 28:53–58, 1974.

## Limitations

Not Applicable

## Ethics authors statements

The platforms’ data redistribution policies were complied with.

## Funding statement

This research received no external funding.

## Institutional review board statement

Not applicable.

## CRediT author statement

**K.R.K.**: Conceptualization; Investigation; Validation; Methodology; Data Interpretation and Visualization; Writing original draft; Manuscript review and Editing; Supervision. **D.B.:** Conceptualization; Investigation; Validation; Data Interpretation and Visualization; Writing original draft; Manuscript review and Editing; Supervision, Project administration and Funding acquisition. **A.S., D.M.**: Conceptualization; Investigation; Methodology; Experimentation; Validation; Data Interpretation and Visualization; Writing original draft; Manuscript review and Editing; Data Interpretation; Resource management for the project. All authors have read and agreed to the published version of the manuscript.

## Declaration of competing interest

The authors declare that they have no known competing financial interests or personal relationships that could have appeared to influence the work reported in this paper.

## Data Availability

No data was used for the research described in the article.
